# Refined secondary Bjerknes force equation for double bubbles with pulsation, translation, and deformation

**DOI:** 10.1016/j.ultsonch.2024.106756

**Published:** 2024-01-09

**Authors:** Juanxiu Liu, Xueping Wang, Jinfu Liang, Yupei Qiao

**Affiliations:** School of Physics and Electronic Science, Guizhou Normal University, Guiyang 550025, China

**Keywords:** Double cavitation bubble, Secondary Bjerknes force, Multiple movement

## Abstract

The secondary Bjerknes force (SBF) is the time-averaged interaction between two bubbles driven in a sound field. We derived a refined formula for the interaction force, incorporating the radial vibration and translational and deformational motions of the bubble. The coupling of pulsation, translation, and deformation enhances the interaction between bubbles but also weakens their stability, making it easier for bubbles to merge or break during motion. The effects of the coupling mode on the magnitude and direction of SBFs coupled with pulsation, translation, and deformation were numerically analyzed and studied. Under certain sound-field conditions, the SBF increased with increasing pressure amplitude, initial radius, and initial velocity, while decreased as the distance increased. In addition, the SBF irregularly increased with increasing frequency.

## Highlights

• Refined secondary Bjerknes forces (SBF) equation for between double cavitation bubbles with pulsation, translation and deformation.
• The coupling of pulsation, translation, and deformation enhances the interaction between two bubbles but also weakens their stability.
• The SBF increases with increasing pressure amplitude, initial radius, initial velocity, while decreases as the distance increases.
• The SBF increased irregularly with increasing frequency.

## 1 Introduction

The phenomenon of sound-field-generated microscopic bubbles (cavitation nuclei) visible to the naked eye is called acoustic cavitation, and the resulting bubbles are known as cavitation bubbles [1]. The radiation pressure generated by other bubbles can cause mutual attraction or repulsion when cavitation bubbles oscillate in an acoustic field [2], [3], [4]. The resultant force is known as the secondary Bjerknes force (SBF) [5], [6], [7], [8], [9].

In 1974, Crum [1] measured the SBF between two pulsating coupled bubbles and discovered that it was much smaller than the main Bjerknes force and buoyancy force based on the linear theory, which was in good agreement with the theory. In 1984, Zabolotskaya [10] used a linear model of the coupled vibration of two bubbles to theoretically prove that the direction of force depends only on the distance between the bubbles. Furthermore, the nonlinear theory of the SBF has been theoretically and experimentally studied. In 1990, Oguz et al. [11] numerically simulated a nonlinear model of two vibrating and shifting bubbles and showed that adding sound waves could reverse the direction of the force. In 1997, Mettin et al. [12] numerically investigated the relationship between the initial separation distance and the bubble size in a robust acoustic field to better understand the SBF.

Recently, Ma et al. [13] calculated the SBF between two bubbles considering bubble deformation. They discovered that the SBF on the two deformed bubbles was significantly higher than that on two spherical bubbles under the same conditions when the bubbles steadily oscillated in the sound field. Zhang et al. [14] investigated the influence of translational motion on the SBF between two oscillating bubbles and between two bubbles with tiny translational motions and found that the SBFs of bubbles with larger translational motions are increased.

Both theory and experiments have demonstrated that multiple movement modes are involved in the actual movement of bubbles, including pulsation, translation, and deformation [15], [16], [17], [18], [19]. The multiple movement modes has a great influence on the interaction between the bubbles [20], [21], [22], [23]. However, to the best of our knowledge, an equation coupling the pulsation, translation, and deformation of two bubbles to describe the interaction between two bubbles has not been reported in the literature. In this study, the interaction between two bubbles considering pulsation, translation, and deformation was investigated under certain sound-field conditions to understand the complex motions of cavitation bubbles and the formation of bubble structures in an ultrasound field.

## 2 Mathematical model

Considering two bubbles with pulsation, translation, and deformation in an ideal incompressible liquid, the velocity potential at position $r_{i}$ from the center of the $i^{\mathrm{th}}$ bubble can be expressed as [24]:

(1) $$\phi_{i}\approx \frac{A_{0}}{r_{i}}P_{0}(\mu_{i})+\frac{A_{1}}{r_{i}^{2}}P_{1}(\mu_{i})+∊\frac{A_{2}}{r_{i}^{3}}P_{2}(\mu_{i}),$$

where $P_{n}(\mu )$is the Legendre polynomial of *n*order, ∊ is a small parameter, which is set to be less than 1 to guarantee the aspherical nature of bubble, and $\mu_{i}=\text{cos}\theta_{i}$, *i* = 1, or 2. Based on the equation for the normal velocity potential at the surface of the $i^{\mathrm{th}}$ bubble [25], [15],

(2) $$\frac{\partial F_{i}}{\partial t}-{\overset{˙}{x}}_{i}\cos\theta_{i}+(\nabla \phi_{i})\cdot e_{r}\frac{\partial F_{i}}{\partial r}=0,\;\mathrm{at}\;r_{i}=S(\theta_{i},t),$$

where $F_{i}\equiv F_{i}(r_{i},\theta_{i},t)=r_{i}-S(\theta_{i},t)$ and $S(\theta_{i},t)\approx R_{i}+∊a_{i}P_{2}(\mu_{i})$ , ∇, and $e_{r}$ represent the gradient with regard to *r* and the normal velocity unit vector, respectively, and ${\overset{˙}{x}}_{i}$represents the translation speed of the $i^{\mathrm{th}}$ bubble center.

Substituting Eq. (1) into Eq.(2)then expanding Eq.(2) with respect to ∊gives

(3) $${∊}^{0}:A_{0}=-R_{i}^{2}\overset{˙}{R_{i}},$$

(4) $$A_{1}=-\frac{1}{2}R_{i}^{3}{\overset{˙}{x}}_{i},$$

(5) $${∊}^{1}:A_{2}=-\frac{1}{3}R_{i}^{3}\left(R_{i}{\overset{˙}{a}}_{i}+2a_{i}{\overset{˙}{R}}_{i}+3a_{i}{\overset{˙}{x}}_{i}P_{1}\right)\text{.}$$

Liquid motion is given by

(6) $$\rho \frac{\partial u_{i}(t)}{\partial t}=-\nabla p_{i}(r_{i},t),$$

where $\nabla p_{i}(r_{i},t)$ is the pressure gradient emitted by the $i^{\mathrm{th}}$ bubble and $u_{i}(t)$ is the velocity field of the $i^{\mathrm{th}}$ bubble, which is expressed as

(7) $$u_{i}(t)=-\nabla \phi_{i}\text{.}$$

The momentary force of the $j^{\mathrm{th}}$ bubble subjected to the $i^{\mathrm{th}}$ bubble is expressed as [26]

(8) $$F_{\mathrm{ij}}=-V_{j}(t)\nabla p_{i}(r_{i},t),$$

where *j* = 1, or, 2, and $j\;\neq \;i,V_{j}(t)$ is the instantaneous volume of the $j^{\mathrm{th}}$ bubble, that is [27]

(9) $$\begin{array}{cc} V_{j}(t) & =\int_{0}^{2\pi }d\varphi \int_{-1}^{1}d\mu_{j}\int_{0}^{r_{j}}r^{2}dr\\ & \approx \frac{4\pi }{3}R_{j}^{3}\left(1+{∊}^{2}\frac{3a_{j}^{2}}{5R_{j}^{2}}\right)\text{.} \end{array}$$

where $r_{j}\approx R_{j}+∊a_{j}P_{2}(\mu_{j})$ denotes the perturbed surface of the $j^{\mathrm{th}}$ bubble, and $P_{2}$=$\frac{1}{2}$(3$\mu_{j}^{2}$-1) is the second-order Legendre polynomials with $\mu_{j}$=$\cos\theta_{j}$.

Based on Eqs. (1), (2), (3), (4), (5), (6), (7), (8), (9)), and by integrating with respect to time over one period, the SBF $\left(F_{Bij}\right)$ of two bubbles with pulsation, translation, and deformation is given by

(10) $$\begin{array}{cc} F_{Bij} & =<F_{ij}>\\ & =-\frac{\rho }{4\pi D^{2}}<-\frac{16\pi^{2}}{3}\left(2R_{i}{\overset{˙}{R}}_{i}^{2}+R_{i}^{2}{\overset{¨}{R}}_{i}+\frac{3R_{i}^{2}{\overset{˙}{R}}_{i}{\overset{˙}{x}}_{i}+R_{i}^{3}{\overset{¨}{x}}_{i}}{D}+\right)\\ & \frac{3P_{2}R_{i}^{2}{\overset{˙}{R}}_{i}\left(R_{i}{\overset{˙}{a}}_{i}+2a_{i}{\overset{˙}{R}}_{i}+3a_{i}{\overset{˙}{x}}_{i}\right)}{D^{2}}+\\ & \left(\frac{P_{2}R_{i}^{3}\left(3{\overset{˙}{a}}_{i}{\overset{˙}{R}}_{i}+3{\overset{˙}{a}}_{i}{\overset{˙}{x}}_{i}+R_{i}{\overset{¨}{a}}_{i}+2a_{i}{\overset{¨}{R}}_{i}+3a_{i}{\overset{¨}{x}}_{i}\right)}{D^{2}}\right)\times \\ & R_{j}^{3}\left(1+\varepsilon^{2}\frac{3a_{j}^{2}}{5R_{j}^{2}}\right)>e_{r}\text{.} \end{array}$$

where $\langle \cdot \rangle$ denotes the time-averaged value of the sound period. Eq. (10) simplifies to the SBF calculated by Mettin [12] when translation and deformation are ignored, which are related to *x* and *a* in Eq.(10), respectively. If cavitation bubble deformation is considered without translation, the SBF is similar to that in Ref. [13]. Meanwhile, if the translation of the cavitation bubbles is considered without deformation, the SBF is similar to that in Ref. [14]. This illustrates that Eq.(10) extends the meaning of SBF between double bubbles in sound field.

To obtain a better understanding of the SBF between two bubbles with pulsation, translation, and deformation, we defined $C_{B}$as in Ref. [13]:

(11) $$\begin{array}{cc} C_{B} & =-\frac{16\pi^{2}}{3}\left(2R_{i}{\overset{˙}{R}}_{i}^{2}+R_{i}^{2}{\overset{¨}{R}}_{i}+\frac{3R_{i}^{2}{\overset{˙}{R}}_{i}{\overset{˙}{x}}_{i}+R_{i}^{3}{\overset{¨}{x}}_{i}}{D}+\right)\\ & \frac{3P_{2}R_{i}^{2}{\overset{˙}{R}}_{i}\left(R_{i}{\overset{˙}{a}}_{i}+2a_{i}{\overset{˙}{R}}_{i}+3a_{i}{\overset{˙}{x}}_{i}\right)}{D^{2}}+\\ & \left(\frac{P_{2}R_{i}^{3}\left(3{\overset{˙}{a}}_{i}{\overset{˙}{R}}_{i}+3{\overset{˙}{a}}_{i}{\overset{˙}{x}}_{i}+R_{i}{\overset{¨}{a}}_{i}+2a_{i}{\overset{¨}{R}}_{i}+3a_{i}{\overset{¨}{x}}_{i}\right)}{D^{2}}\right)\times \\ & R_{j}^{3}\left(1+\varepsilon^{2}\frac{3a_{j}^{2}}{5R_{j}^{2}}\right) \end{array}$$

when $\langle C_{B}\rangle >0$, the SBF is attractive; otherwise, it is repulsive.

In Eqs. (10), (11), the values of $R_{i},{\overset{˙}{R}}_{i},x_{i},{\overset{˙}{x}}_{i},a_{i}$, and ${\overset{˙}{a}}_{i}$ can be obtained by numerically solving Eqs. (12), (13), (14), (15), (16), (17), (18), (19), (20), coming from Ref. [15], are derived to describe the pulsation, translation, and deformation of two coupling bubble in an sound field. The radial oscillation, translation, and deformation of the $i^{\mathrm{th}}$ bubble are defined as

(12) $$\begin{array}{cc} {∊}^{0}:\; & R_{i}{\overset{¨}{R}}_{i}+\frac{3}{2}{\overset{˙}{R}}_{i}^{2}-\frac{1}{4}{\overset{˙}{x}}_{i}^{2}-{\overset{˙}{B}}_{0}-\frac{3}{4}B_{1}^{2}+\\ & \frac{3}{2}B_{1}{\overset{˙}{x}}_{i}-\frac{p_{i}(r)-p_{\infty }}{\rho }=0 \end{array}$$

(13) $$\frac{3}{2}{\overset{˙}{R}}_{i}{\overset{˙}{x}}_{i}+\frac{1}{2}R_{i}{\overset{¨}{x}}_{i}-\frac{3}{2}R_{i}{\overset{˙}{B}}_{1}-\frac{3}{2}{\overset{˙}{R}}_{i}B_{1}=0,$$

and

(14) $$\begin{array}{cc} {∊}^{1}:\; & \frac{1}{3}R_{i}{\overset{¨}{a}}_{i}-\frac{1}{3}a_{i}{\overset{¨}{R}}_{i}-\frac{2}{R_{i}}\left(a_{i}{\overset{˙}{x}}_{i}^{2}+a_{i}B_{1}^{2}\right)-\\ & \frac{5}{3}R_{i}^{2}{\overset{˙}{B}}_{2}-\frac{10}{3}B_{2}R_{i}{\overset{˙}{R}}_{i}+{\overset{˙}{a}}_{i}{\overset{˙}{R}}_{i}+\frac{4a_{i}{\overset{˙}{x}}_{i}B_{1}}{R_{i}}=0, \end{array}$$

with

(15) $$B_{0}\approx -\frac{R_{3-i}^{2}{\overset{˙}{R}}_{3-i}}{D}+\frac{R_{3-i}^{3}{\overset{˙}{x}}_{3-i}}{2D^{2}},$$

(16) $$B_{1}\approx -\frac{R_{3-i}^{2}{\overset{˙}{R}}_{3-i}}{D^{2}}+\frac{R_{3-i}^{3}{\overset{˙}{x}}_{3-i}}{D^{3}},$$

(17) $$B_{2}\approx -\frac{R_{3-i}^{2}{\overset{˙}{R}}_{3-i}}{D^{3}}+\frac{R_{3-i}^{3}{\overset{˙}{x}}_{3-i}}{D^{4}}\text{.}$$

where ${\overset{¨}{R}}_{i}$ is the second derivative of $R_{i}$ over time and $p_{i}(r)$is as follow,

(18) $$p_{i}(r)=p_{\mathrm{gi}}-p_{d}-\frac{2\sigma }{R_{i}}-\frac{4\eta }{R_{i}}{\overset{˙}{R}}_{i}+\frac{R_{i}}{c}\frac{d}{\mathrm{dt}}\left(p_{\mathrm{gi}}-p_{d}\right),$$

(19) $$p_{\mathrm{gi}}=\left(p_{0}+\frac{2\sigma }{R_{0i}}\right){\left(\frac{R_{0i}^{3}-h_{i}^{3}}{R_{i}^{3}-h_{i}^{3}}\right)}^{\gamma },$$

(20) $$p_{d}=-P_{a}\sin(2\pi \mathrm{ft})\text{.}$$

where $h_{i}=8.5/R_{0i}$.

## 3 Numerical simulation

In this study, the interactions between two bubbles with pulsation, translation, and deformation were investigated. The SBFs were numerically calculated under different driving pressures, ultrasound frequencies, initial radii,initial distance, and initial translation velocities. Moreover, we used the criterion of the Rayleigh–Taylor instability (RTI) [28] to obtain the stable region in different phase pictures and ensure periodical two-bubble motion. The criterion for the RTI is

(21) $$\underset{t\subset \left[0,N(2\pi /\omega )\right]}{\max}\left|\frac{a_{i}}{R_{i}}\right|⩾1$$

where *N* is an integer greater than zero and $a_{i}$ and $R_{i}$ are obtained by numerically solving Eqs. (12), (13), (14). Table 1 lists the physical parameters utilized in the computations.

**Table 1:** Physical variables incorporated into the computation.

Symbol	Definition (value)
ρ	Density of a liquid ($10^{3}\;\mathrm{kg}/m^{3}$)
η	Liquid’s viscosity coefficient (0.001 Pa·s)
σ	Surface tensile strength (0.0725 N/m)
γ	Bubble gas polytropic index (1.4)
*c*	Velocity of sound (1481 m/s)
$p_{\infty }$	Pressure at the infinity ($1.013\times 10^{5}\;P_{a}$)
$p_{0}$	Ambient pressure ($1.013\times 10^{5}\;P_{a}$)
$P_{a}$	Driving sound pressure amplitude
*f*	Ultrasound frequency ($2.5\times 10^{4}\;\mathrm{Hz}$)
$R_{0\;i}$	Initial radius of the $i^{\mathrm{th}}$ bubble
${\overset{˙}{R}}_{0\;i}$	Initial radius velocity of the $i^{\mathrm{th}}$ bubble (0)
$x_{0\;i}$	Initial displacement of the $i^{\mathrm{th}}$ bubble
${\overset{˙}{x}}_{0\;i}$	Initial translation velocity of the $i^{\mathrm{th}}$ bubble
$a_{0\;i}$	Initial deformation of the $i^{\mathrm{th}}$ bubble (0)
${\overset{˙}{a}}_{0\;i}$	Initial deformation velocity of the $i^{\mathrm{th}}$ bubble (0)
$F_{B}$	The SBF of two bubbles coupled with pulsation, translation, and deformation
$F_{B}^{\prime }$	The SBF of two bubbles coupled with pulsation and translation
$F_{B}^{\prime \prime }$	The SBF of two bubbles coupled with pulsation and deformation

### 3.1 Effect of driving pressure amplitude

**Fig. 1:**
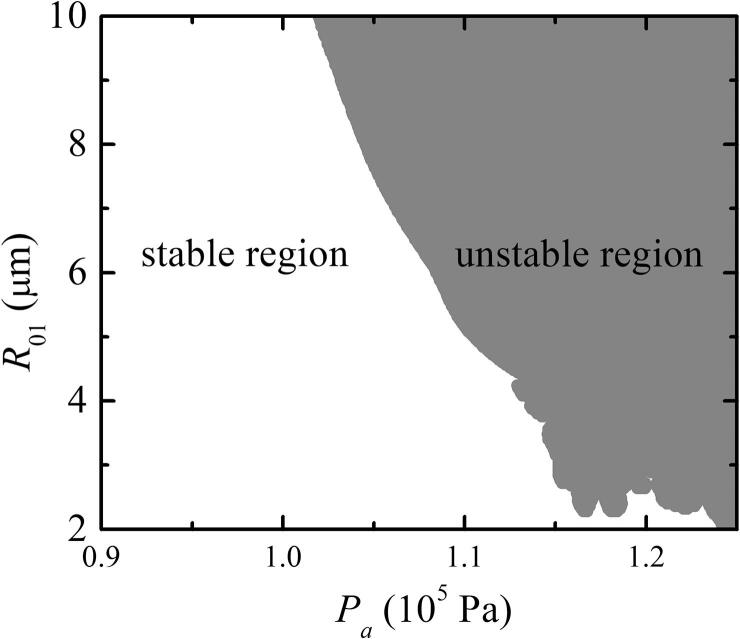
Phase diagram of double-bubble system with pulsation, translation, and deformation. The gray and white regions show the unstable and stable shapes, respectively. ${\overset{˙}{R}}_{01}=0,R_{02}=4\;\mu \text{m},{\overset{˙}{R}}_{02}=0,x_{01}=0,x_{02}=500\;\mu \text{m},{\overset{˙}{x}}_{01}=0$, and ${\overset{˙}{x}}_{02}=0$.

Based on Eqs. (12), (13), (14), the $P_{a}$-$R_{01}$phase diagram of two bubbles coupled with pulsation, translation, and deformation can be obtained (Fig. 1). The white region denotes the stable-shape region of the two-bubble oscillation, whereas the gray region represents the unstable-shape region. In this study, we considered only the SBF between two bubbles within a stable region, as shown in Fig. 1.

**Fig. 2:**
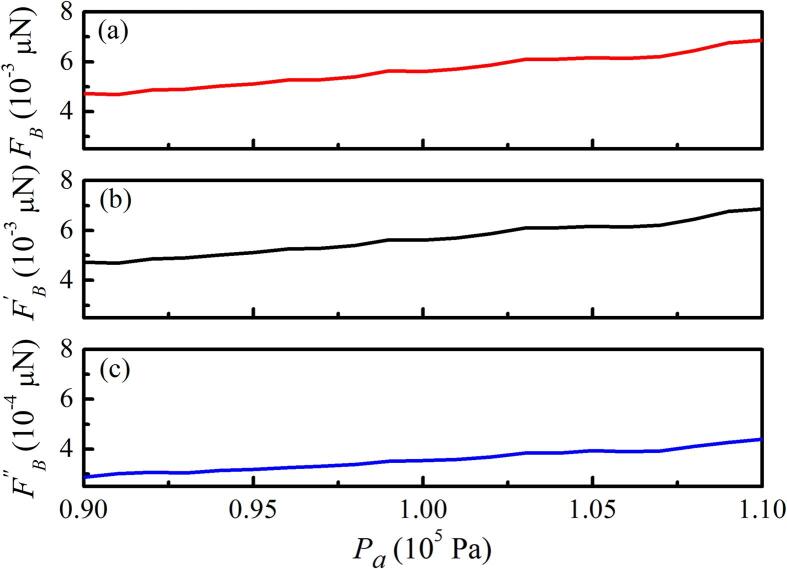
SBF between two bubbles under different driving pressure amplitudes. $R_{01}=2\;\mu \text{m},{\overset{˙}{R}}_{01}=0,R_{02}=4\;\mu \text{m},{\overset{˙}{R}}_{02}=0,x_{01}=0,x_{02}=500\;\mu \text{m},{\overset{˙}{x}}_{01}=0$, and ${\overset{˙}{x}}_{02}=0$. (a) Two bubbles with pulsation, translation, and deformation; (b) two bubbles with pulsation and translation; (c) two bubbles with pulsation and deformation.

Fig. 2(a) shows the SBF between two bubbles with pulsation, translation, and deformation, based on Eq. (10). As shown in Fig. 2(a) the interaction between the two bubbles increased with the driving pressure amplitude. We calculated the SBF between two bubbles with only pulsation and translation [Fig. 2(b)] and with only pulsation and deformation [Fig. 2(c)] to further investigate the coupling effect of pulsation, translation, and deformation on the interaction between two bubbles. By comparing Fig. 2(a)–(c), although the absolute value of the SBF between two bubbles increases with the sound pressure amplitude, the SBF between two bubbles with pulsation, translation, and deformation is approximately 10 times greater than that with only pulsation and deformation, and almost coincides with the SBF with only pulsation and translation.

### 3.2 Effect of initial radius

**Fig. 3:**
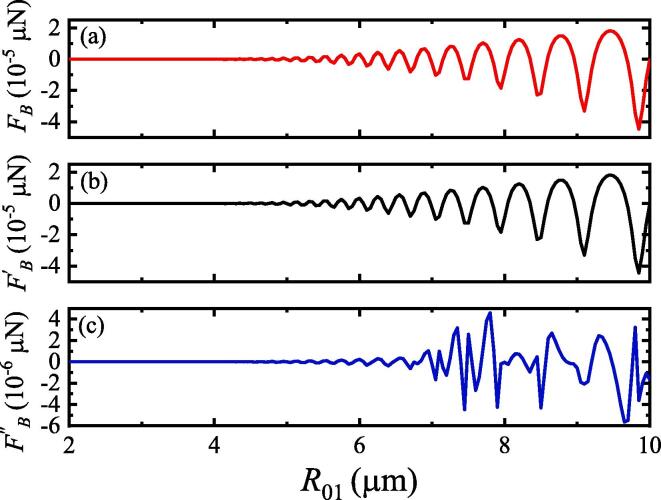
SBF between two bubbles under different initial radii. $P_{a}=1.1\times 10^{5}$ Pa, ${\overset{˙}{R}}_{01}=0,R_{02}=2\;\mu \text{m},{\overset{˙}{R}}_{02}=0,x_{01}=0,x_{02}=500\;\mu \text{m},{\overset{˙}{x}}_{01}=0$, and ${\overset{˙}{x}}_{02}=0$. (a) Two bubbles with pulsation, translation, and deformation; (b) two bubbles with pulsation and translation; (c) two bubbles with pulsation and deformation.

We calculated the SBF between the two bubbles using Eq. (10) to investigate the effect of the initial radius on the SBF between the two bubbles with pulsation, translation, and deformation. Fig. 3 shows that the SBF increases with increasing initial radius for the three motion modes of the two bubbles. However, the SBF between the two bubbles with pulsation, translation, and deformation [Fig. 3(a)] is larger than that with only pulsation and translation [Fig. 3(b)] or only pulsation and deformation [Fig. 3(c)], demonstrating that the effect of coupling pulsation, translation, and deformation on the interaction between the two bubbles is stronger than that with only pulsation and translation or only pulsation and deformation.

### 3.3 Effect of driving frequency

**Fig. 4:**
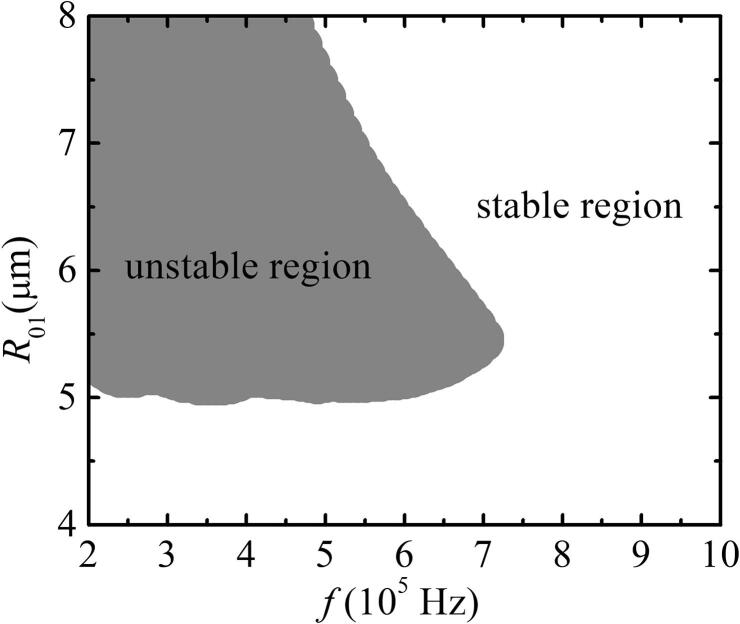
Phase diagram of double-bubble system coupling pulsation, translation, and deformation. $P_{a}=1.1\times 10^{5}$ Pa, ${\overset{˙}{R}}_{01}=0,R_{02}=4\;\mu \text{m},{\overset{˙}{R}}_{02}=0,x_{01}=0,x_{02}=500\;\mu \text{m},{\overset{˙}{x}}_{01}=0$, and ${\overset{˙}{x}}_{02}=0$.

Fig. 4 shows the phase diagram of the driving frequency *f* and initial radius $R_{01}$ for two bubbles with pulsation, translation, and deformation. The white and gray regions in Fig. 4 are stable and unstable regions, respectively.

**Fig. 5:**
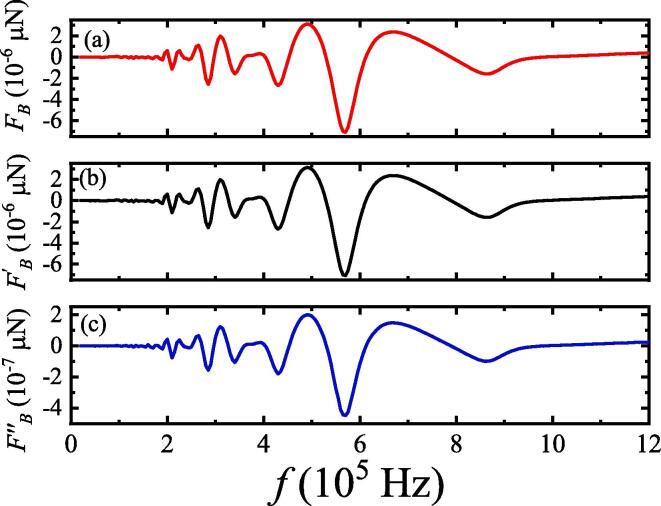
SBF between two bubbles under different ultrasound frequencies. $P_{a}=1.1\times 10^{5}$ Pa, $R_{01}=2\;\mu \text{m},{\overset{˙}{R}}_{01}=0,R_{02}=4\;\mu \text{m},{\overset{˙}{R}}_{02}=0,x_{01}=0,x_{02}=500\;\mu \text{m},{\overset{˙}{x}}_{01}=0$, and ${\overset{˙}{x}}_{02}=0$. (a) Two bubbles coupling pulsation, translation and deformation; (b) two bubbles coupling pulsation and translation; (c) two bubbles coupling pulsation and deformation.

In the stable region,we calculated the SBF between two bubbles with different modes of motion based on Eq. (10). Fig. 5 shows that the maximum magnitude of SBF between two bubbles is at a frequency of $5.75\times 10^{5}\mathrm{Hz}$. This may be because of the motion of bubbles corresponding to the resonant frequency of the two-bubble system [29]. In addition,the SBF between two bubbles with pulsation,translation,and deformation [Fig. 5(a)] fluctuates more than that of two bubbles with only pulsation and translation [Fig. 5(b)] or only pulsation and deformation [Fig. 5(c)].

### 3.4 Effect of initial translational velocity

**Fig. 6:**
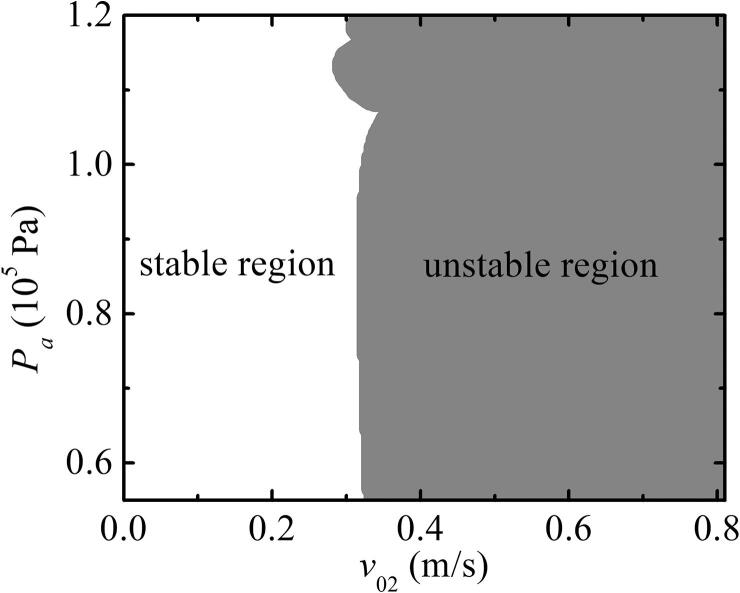
Phase diagram of a bubble for the secondary shape mode. $R_{01}=2\;\mu \text{m},{\overset{˙}{R}}_{01}=0,R_{02}=4\;\mu \text{m},{\overset{˙}{R}}_{02}=0,x_{01}=0,x_{02}=500\;\mu \text{m}$, and ${\overset{˙}{x}}_{02}=0$.

Fig. 6 shows the phase diagram of $v_{02}-P_{a}$ for two bubbles with pulsation, translation, and deformation. The white and gray regions in Fig. 6 represent the stable and unstable regions, respectively. In the stable region, we calculated the SBF between the two bubbles based on Eq. (10).

**Fig. 7:**
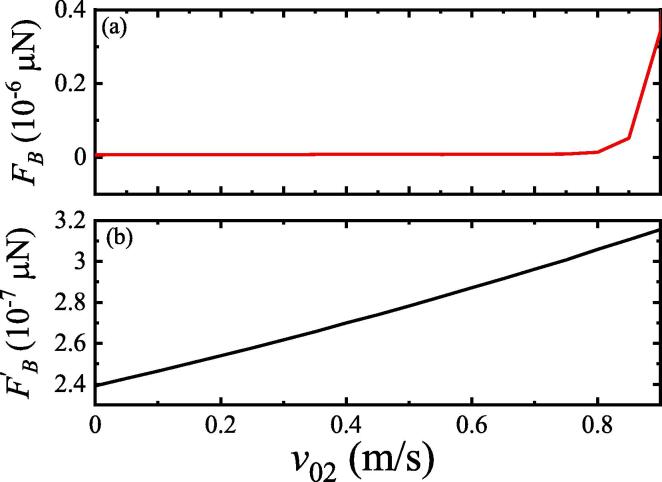
SBF between two bubbles under different translation initial velocities.$P_{a}=1.13\times 10^{5}$ Pa, $R_{01}=2\;\mu \text{m},{\overset{˙}{R}}_{01}=0,R_{02}=4\;\mu \text{m},{\overset{˙}{R}}_{02}=0,x_{01}=0,x_{02}=500\;\mu \text{m}$, and ${\overset{˙}{x}}_{02}=0$. (a) Two bubbles coupling pulsation, translation, and deformation; (b) two bubbles coupling pulsation and translation.

Fig. 7 shows that the SBF between the two bubbles increased as the initial velocity increased. However, the SBF between the two bubbles with pulsation, translation, and deformation [Fig. 7(a)] was slightly larger than that with pulsation and translation [Fig. 7(b)]. The force sharply increased when the velocity approached 1m/s. This may be because the deformation parameters of the bubble are small compared with the pulsation and translation of the bubble; thus, the effect on the interaction between the two bubbles is relatively weak.

### 3.5 Effect of distance

**Fig. 8:**
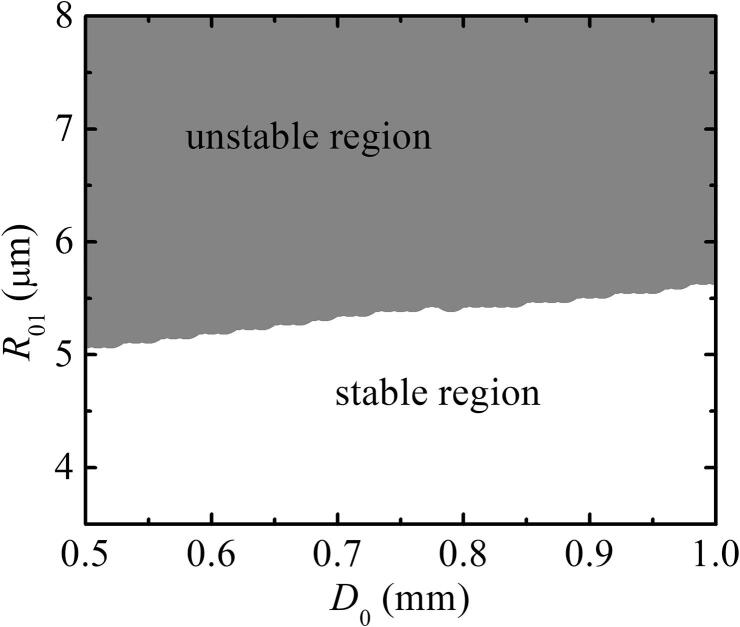
Phase diagram of double-bubble system coupling pulsation, translation, and deformation. $P_{a}=1.1\times 10^{5}$ Pa, ${\overset{˙}{R}}_{01}=0,R_{02}=4\;\mu \text{m},{\overset{˙}{R}}_{02}=0,x_{01}=0,{\overset{˙}{x}}_{01}=0$, and ${\overset{˙}{x}}_{02}=0$.

The distance between two bubbles is an important factor affecting SBF. For the two bubbles with pulsation, translation, and deformation, the distance, $D=|x_{i}-x_{j}|$, between bubbles varies with time. The $x_{i}$ and $x_{j}$ are the displacements of two bubbles center, respectively. If the initial value of $x_{i}$ is set to be zero, the initial value of $x_{j}$ is the initial distance($D_{0}$) between two bubbles. Fig. 8 shows the phase diagram of $D_{0}-R_{01}$ for two bubbles with pulsation, translation, and deformation. The white and gray regions in Fig. 8 are stable and unstable regions, respectively.

**Fig. 9:**
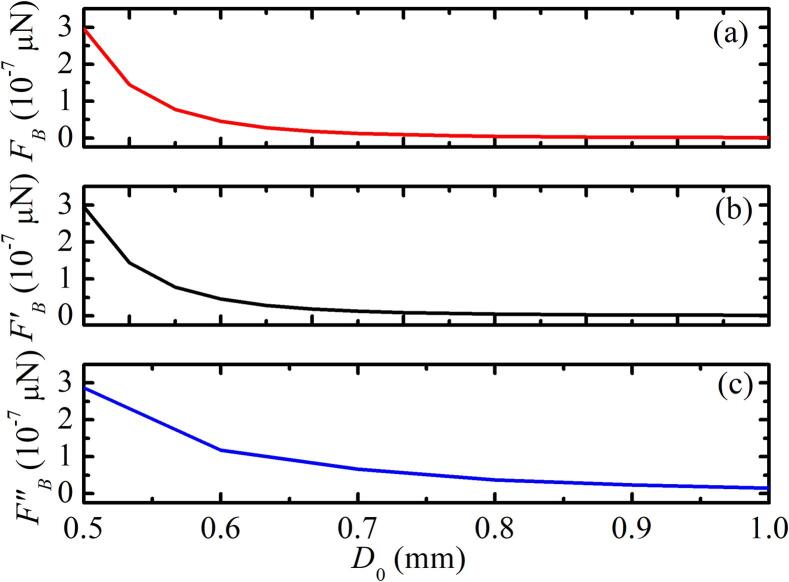
SBF between two bubbles under different initial distances. $P_{a}=1.15\times 10^{5}$ Pa, $R_{01}=2\;\mu \text{m},{\overset{˙}{R}}_{01}=0,R_{02}=4\;\mu \text{m},{\overset{˙}{R}}_{02}=0,x_{01}=0,{\overset{˙}{x}}_{01}=0$, and ${\overset{˙}{x}}_{02}=0$. (a) Two bubbles coupling pulsation, translation and deformation; (b) two bubbles coupling pulsation and translation; (c) two bubbles coupling pulsation and deformation.

We choose the different $D_{0}$ in the stable region to numerically calculate based on Eqs. (12), (13), (14) and (10) to analyze the influence of the distance on the second Bjerknes force(SBF). Fig. 9 illustrates that the SBF between bubbles decreases as the distance increases. If the distance is sufficiently large, the two bubbles are not coupled and the SBF is close to zero, as shown in Fig. 9. However, for two bubbles coupled with pulsation, translation and deformation, the results show that the SBF has a larger interference distance than the SBF of pulsation and translation or pulsation and deformation.

## 4 Conclusions

The equation describing the SBF among the double-bubble coupling pulsation, translation, and deformation was obtained using the velocity potential and perturbation theory. The SBF between two bubbles was numerically calculated under different sound pressure amplitudes, initial radii, ultrasound frequencies, and initial translation velocities. In the stable region, the SBF between two bubbles increases as the sound pressure amplitude and initial radius increase, but it decreases with increasing initial translation velocity because the distance between the two bubbles increases. The SBF between the two bubbles did not increase with increasing frequency because of the resonant frequency of the two-bubble system.The SBF between two bubbles gradually decreases as the distance increases, and if the distance is sufficiently large, the bubbles are no longer coupled and the SBF is close to zero. In addition, the SBF between two bubbles coupling pulsation, translation, and deformation was stronger than that between two bubbles with pulsation and translation or with pulsation and deformation. This study is conducive to further understanding the dynamics of two-bubble systems and the formation of acoustic cavitation bubble structures.

## CRediT authorship contribution statement

**Juanxiu Liu:** Conceptualization, Software, Visualization, Writing – original draft. **Xueping Wang:** Writing – review & editing. **Jinfu Liang:** Methodology, Funding acquisition, Supervision. **Yupei Qiao:** Methodology, Formal analysis.

## Declaration of competing interest

The authors declare that they have no known competing financial interests or personal relationships that could have appeared to influence the work reported in this paper.
